# Physical Activity Outcomes of a Culturally Tailored, Father-Focused, and Family-Centered Health Promotion Program for Mexican-Heritage Families: *¡Haz Espacio Para Papi!* (Make Room for Daddy)

**DOI:** 10.3390/ijerph21111475

**Published:** 2024-11-06

**Authors:** M. Renée Umstattd Meyer, Tyler Prochnow, Marilyn E. Wende, Kelly R. Ylitalo, Rodney X. Sturdivant, Cassandra M. Johnson, Haley Delgado, Stewart G. Trost, Luis Gómez, Joseph R. Sharkey

**Affiliations:** 1 Department of Public Health, Robbins College of Health and Human Sciences, Baylor University, Waco, TX 76798, USA; tprochnow@tamu.edu (T.P.); kelly_ylitalo@baylor.edu (K.R.Y.); delgado_h@heritage.edu (H.D.); 2 Department of Health Behavior, School of Public Health, Texas A&M University, College Station, TX 77843, USA; 3 Department of Health Education & Behavior, University of Florida, Gainesville, FL 32608, USA; marilyn.wende@ufl.edu; 4 Department of Statistical Sciences, Baylor University, Waco, TX 76798, USA; rodney_sturdivant@baylor.edu; 5 Nutrition and Foods Program, School of Family and Consumer Sciences, Texas State University, San Marcos, TX 78666, USA; cassandra_johnson@txstate.edu; 6 Department of Nursing, Heritage University, Toppenish, WA 98948, USA; 7 School of Human Movement and Nutrition Sciences, The University of Queensland, Brisbane, QLD 4072, Australia; s.trost@uq.edu.au; 8 Department of Environmental & Occupational Health, School of Public Health, Texas A&M University, College Station, TX 77843, USA; gomez@tamu.edu; 9 School of Public Health, Texas A&M University, College Station, TX 77843, USA; jrsharkey@tamu.edu

**Keywords:** sedentary behavior, *promotora*, community health worker, low-income, intervention, border health

## Abstract

Despite the health benefits of physical activity (PA), many individuals do not meet PA recommendations. Family-centered PA approaches, particularly active engagement by Mexican-heritage fathers, may support family PA. This study reports PA outcomes of a culturally tailored, father-focused, and family-centered, program for Mexican-heritage families. *Promotora* researchers recruited participating families (n = 59, n = 42 complete cases), consisting of children (mean age: 10.1 [SD = 0.9]), fathers, and mothers from five randomly selected geographic clusters in low-resourced *colonias* in south Texas, in a stepped-wedge randomized design. PA was measured using wrist-worn ActiGraph GT9X accelerometers. Statistical analyses for moderate-to-vigorous PA (MVPA), light PA (LPA), and sedentary time for the child, father, and mother were conducted using linear mixed models. The findings were as follows: children had no significant changes in MVPA (*p* = 0.18), LPA (*p* = 0.52), or sedentary behavior (*p* = 0.74); fathers had no significant changes in MVPA (*p* = 0.94), LPA (*p* = 0.17), or sedentary behavior (*p* = 0.15); and mothers had a *significant* decrease in LPA (*p* < 0.01), and no significant changes in MVPA (*p* = 0.66) or sedentary behavior (*p* = 0.77). Despite null results, this study provides an example of a culturally tailored, family-focused program implemented among Mexican-heritage families with limited PA resources and opportunities. Future PA interventions may require higher PA-focused doses over longer time periods to produce a significant change in LPA, MVPA, or sedentary time.

## 1. Introduction

Physical activity (PA) is consistently supported as a preventative strategy to decrease the risk of chronic diseases and promote healthy physical and mental functioning [1]. PA is associated with improved quality of life, mental health, and cognition [2]. While there are benefits to PA across the lifespan, only 26.1% of children [3] and 23% of adults in the United States (U.S.) [4,5] meet PA guidelines. PA guidelines suggest that children participate in at least 60 min of daily PA, including bone and muscle-strengthening activities [6,7]. Adults are recommended to attain 150–300 min of moderate intensity or 75–150 min of vigorous intensity PA (or a combination of the two), and to engage in muscle-strengthening PA weekly [6,7]. Guidelines also suggest that adults and children reduce time spent doing sedentary behaviors [6,7]. Public health interventions to increase adherence to PA guidelines are increasingly prioritizing populations and communities with limited access to health-promoting resources to offer more PA opportunities [8,9,10].

A subpopulation of individuals who face substantial barriers to PA participation and chronic disease prevention are Mexican-heritage families who reside in *colonias* in the Lower Rio Grande Valley on the U.S.–Mexico border. *Colonias* along the Texas border are systematically underserved due to their lack of access to basic infrastructure (i.e., drinking water, sewage treatment, paved roads), their isolated geographic locations, high poverty rates, lack of access to quality health care and health promotion initiatives, and disproportionate rates of chronic conditions [11,12]. *Colonias* are home to approximately 500,000 people, 96% of whom who identify as Hispanic or Latino/a [13,14,15]. An estimated 23.9% of *colonia* residents live in poverty, rates which are substantially higher than those of Texas (14.2%) and the U.S. (11.6%) [16]. Few *colonias* have PA resources like parks and other public spaces for residents to be active [13,17,18]. Research conducted among adults living in *colonias* suggests low adherence to PA guidelines, with 67.6% of Hispanic respondents who reside in *colonias* not meeting PA recommendations, compared to 55.6% of Hispanic respondents residing in other locations in the U.S. [19].

Family and cultural values play an important role in health behaviors, including PA. For example, Latino families often report high levels of collectivism and familism [20,21,22,23]. Collectivism is defined as providing financial or social support to one’s familial unit above all else [24]. Similarly, familism is an emphasis on having loyalty and pride in one’s family [25,26]. PA of Mexican-heritage fathers residing in *colonias* is associated with the number of familial social connections [27], and research on Mexican-heritage sibling dyads shows that younger siblings may emulate the PA behavior of their older siblings [28]. To address increased barriers to PA and rates of chronic conditions in *colonias*, it is crucial to apply culturally tailored health promotion programs [29,30]. Mexican-heritage child PA is associated with frequency of active play with close social contacts, which can be modified through intervention [31,32]. Hence, applying a family systems approach may improve PA [33,34]. Family systems theory posits that a family functions as a system, wherein people are expected to interact with and respond to one another in certain ways, thus creating family norms and social influence on health behavior [35]. A family systems approach to PA promotion includes increased benefits for PA outcomes compared to individual interventions, likely due to improved role modeling and opportunities [36].

Recent literature on family-centered and culturally adapted PA interventions has shown promising results in various populations (e.g., primary school-aged daughters and preschoolers) [37,38,39], with studies by O’Connor et al. [40,41] and Perez et al. [42] demonstrating the feasibility and acceptability of culturally adapted, father-focused obesity interventions for Hispanic families. Few family-centered programs have included adequate engagement of fathers [43,44]. Recent research has recognized the specific impact of Latino fathers on the health and wellbeing of their children [43,45]. While there have been notable father-focused program implementations (e.g., the “Healthy Dads, Healthy Kids” obesity treatment program in Australia) [46,47], additional research is needed on culturally tailored programs of this kind [41]. Only one study using a family-centered program and incorporating the father’s role in promoting health behaviors has occurred in the U.S. among Mexican-heritage populations [41]. However, this study recruited participants in an urban location where resource access may be higher [41]. Mexican-heritage families in *colonias* face unique challenges that may impact PA behaviors. These include limited access to safe recreational spaces, cultural beliefs about PA, and socioeconomic barriers [17]. Understanding these contextual factors is crucial for developing effective, tailored interventions for this population.

To fill the gap on engaging fathers in health programs and provide opportunities and resources for PA, the purpose of this article is to report the PA outcomes of the *¡Haz Espacio para Papi!* (Make Room for Daddy; HEPP!) culturally tailored, father-focused, and family-centered health promotion program for Mexican-heritage families residing in *colonias* in south Texas.

## 2. Materials and Methods

This manuscript reports on PA outcomes from the HEPP! program. Specifically, the HEPP! program was a 6-week, father-focused, family-centered nutrition and PA program for Mexican-heritage families residing in *colonias* in south Texas along the Lower Rio Grande Valley [48,49,50]. Trained *promotora* researchers contributed to all elements of this program, including community assessments, program design, implementation, evaluation, and data interpretation [49]. *Promotoras* (*promotoras de salud*) are female community health workers who work and live in Latino and Hispanic communities and are actively engaged in coordinating or ensuring health care service provision, health promotion, and outreach [51,52]. In addition to these roles, *promotora* researchers also serve as research collaborators in community–academic partnerships, receive training in research methods, and are key members of the research team [51,52]. Prior to recruitment, all materials were approved by the referent Institutional Review Board (IRB).

### 2.1. Setting and Participants

Families were recruited from *colonias* east of Edinburg, Texas, in areas referred to in this study as the San Carlos area. Prior to the program, *promotora* researchers led formative work within 18 total geographic clusters in Hidalgo County, 12 of which were in the San Carlos area. These 12 were randomized, and the first 5 geographic clusters identified were selected to participate in the HEPP! program. *Promotora* researchers identified potential participants by completing door-to-door canvassing and recontacting the participants from previous studies who consented to recontact. Eligible families met the following criteria: (1) have no food allergies; (2) have no PA restrictions; (3) have a child 9–11 years of age at time of program enrollment; (4) both parents/partners are at least 21 years of age; (5) at least one parent/grandparent/great-grandparent to child participants are of Mexican origin (i.e., Mexican heritage); (6) both parents prefer to speak, write, and read in Spanish; and (7) both parents actively live/reside in the same household.

**Figure 1:**
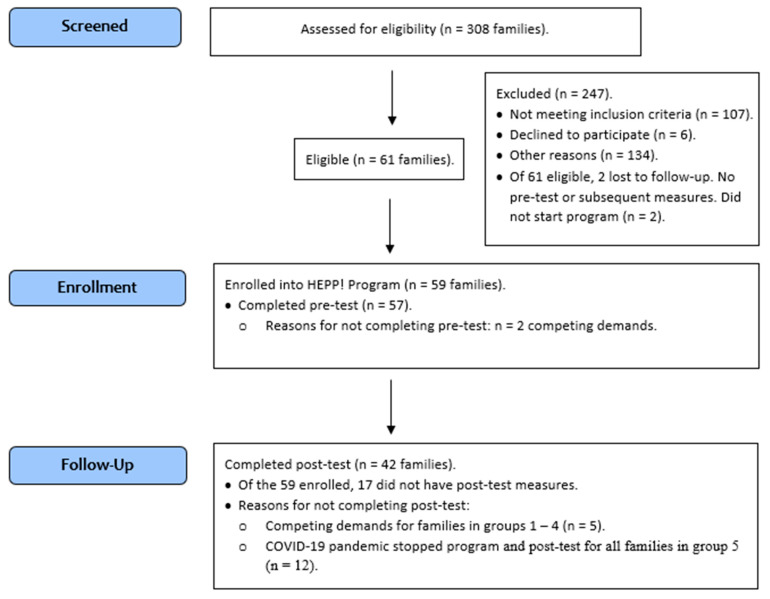
CONSORT Flow Diagram.

*Promotora* researchers screened a total of 308 families across 18 geographic clusters in Hidalgo County. These 18 geographic clusters were randomized. Of these 308 families, 59 were enrolled from the first 5 geographic clusters identified. These 59 families completed baseline (pre-test) measures for the father, mother, and child (see Figure 1). Due to logistical constraints (e.g., facility space needing to meet requirements for nutrition and physical activity, personnel) and to allow for deeper one-on-one interactions between program staff and participating families, only one cluster (9–13 families) completed the program at a time, and families were subdivided into smaller groups of 4–6 families for the Saturday morning or afternoon program sessions. Figure 1 shows the flow of participants from recruitment through enrollment, baseline/pre-test, and post-test measures.

Families completed the program between July 2019 and February 2020. Group 1 families (n = 12 families) participated in the program from July 2019–August 2019; Group 2 (n = 10) was from August 2019–September 2019; Group 3 (n = 13) was from October 2019–November 2019; and Group 4 (n = 12) was from November 2019–January 2020 (to adjust for holidays). Due to the COVID-19 pandemic and changes to face-to-face interactions, Group 5’s (n = 12) participation was truncated, and only their control period data are included, since they did not finish the full program as originally designed and only participated in 2 out of the 6 sessions (February 2020). Five additional families withdrew from the program and did not agree to undergo post-test measurements (see Figure 1, Participant Flowchart), and three families did not complete the program, but agreed to undergo post-test measurements, and were subsequently included in analyses. This analytic sample included 59 families who enrolled in the study; of these, 42 families had complete follow-up measures.

### 2.2. Study Design

Random sampling was used to identify geographic clusters for program participants. Random assignment was not used to allocate families into program and control groups; rather, a modified stepped-wedge, cluster randomized design (quasi-experimental) was used to test program effects between the program and a delayed control, while balancing ethical and practical considerations [53]. Supplementary Information S1 presents the study design.

### 2.3. ¡Haz Espacio Para Papi! (HEPP!) Program

The objectives of the HEPP! program were to improve dietary intake of fruits and vegetables, increase PA, and enhance family functioning among Mexican-heritage family triads (children, fathers, and mothers) residing in *colonias*, with a primary focus on children and fathers. A more detailed description of the HEPP! program, PA curriculum, and process evaluation has been published [48,50]. In brief, six weekly sessions were delivered by *promotora* researchers, held at a local community center, and each lasted roughly 150 min, including all program components (e.g., interactive nutrition and cooking education, PA, family dynamics).

The PA curriculum embraced existing traditions while encouraging new additions to increase active play [48]. For example, several lessons added active variations to traditional games played among Mexican-heritage families. Lessons incorporated modified concepts from SPARK physical education and after school programing [54], and “Healthy Dads, Healthy Kids” [47], with theoretical grounding in the Social Cognitive Theory [55] and Family Systems Theory [35]. Activities were designed to engage the child, father, and mother, but primarily focused on father–child co-participation in light-to-moderate PA. Take-home challenges for the entire family provided continuity between in-person sessions, which were also short in duration, with fun activities. Each week, families were given two at-home challenges to complete between sessions [48].

### 2.4. Measures

All measures were collected in-person by *promotora* researchers. Sociodemographic variables were collected at baseline via surveys, and they included age, sex, and country of birth. Surveys were administered in the preferred language of choice (Spanish or English). The intervention dose was measured as the number of sessions attended, and the number of minutes with a PA-focus across all sessions. The number of sessions attended was enumerated using attendance documents, and categorized (0, 1–3, 4–5, or >5 sessions). The total minutes of PA-focused time across all sessions were calculated by adding the planned PA-focused minutes for each session when the family was in attendance. This summed variable was categorized (0, 1–100, or >100 min).

#### 2.4.1. Body Mass Index (BMI)

Height and weight were collected using a HM200P PortStad portable stadiometer and digital scale at baseline. BMI was calculated for adults using height and weight, and it was calculated as BMI = kg/m^2^, where kg is weight in kilograms and m^2^ is height in meters squared. BMI categories were determined based on recommended cutoffs [56] for underweight (BMI < 18.5 kg/m^2^), normal weight (BMI 18.5 to <25 kg/m^2^), overweight (BMI 25 to <30 kg/m^2^), and obese (BMI ≥ 30 kg/m^2^). BMI-for-age z-scores for children were calculated according to the 2000 CDC Growth Charts for children ages 0 to <20 years [57]. Overweight was defined as a z-score > +1 SD, and obesity was defined as >+2 SD [58].

#### 2.4.2. Device-Measured Physical Activity

ActiGraph GT9X accelerometers (ActiGraph Corporation, Pensacola, FL, USA) were used to measure daily time spent doing sedentary, light PA (LPA), and moderate-to-vigorous PA (MVPA). Fathers, mothers, and participating children were asked to wear the accelerometer on their non-dominant wrist 24 h per day for 7 days at three separate timepoints, as follows: (1) at baseline (prior to being enrolled as the “control” arm); (2) during the transition measurements, (6–8 weeks after baseline data collection); and (3) post-intervention (6–8 weeks after the transition timepoint and 12–16 weeks after baseline). Non-wear periods were identified according to the procedures established by Ahmadi et al. [59], which differentiate non-wear periods from sleep. Sleep periods were detected using the algorithm developed by Van Hees et al. [60], while non-wear periods were identified and differentiated from sleep using the methods described by Ahmadi and colleagues [59]. Monitoring days were considered valid if the wear time was greater than 960 min per day [60]. To be included in the analysis, participants were required to have at least four valid monitoring days, with at least one of those days being a weekend day.

Raw accelerometer data collected at 30 Hz were processed into PA metrics using a machine-learned random forest classifier that was specifically designed and validated for assessing PA in school-aged youth [61,62] and free living adults [63]. The classifier for school-aged children uses features in the raw acceleration signal to identify/quantify time spent doing sedentary activities (sitting/lying down); light-intensity activities and games, such as walking and running; and moderate-to-vigorous intensity activities and games [61,62]. The adult random forest model classified movement behaviors as sedentary (lying/sitting still), stationary plus (active sitting/standing, still/active standing), walking, or running [63]. Further information about machine-learning algorithms and their application to accelerometry can be found elsewhere [64]. For children, MVPA was defined as the sum of daily time spent walking, running, and engaging in moderate-to-vigorous activities and games, and LPA was defined as the sum of daily time spent doing light-intensity activities and games. For adults, MVPA was defined as the sum of daily time spent walking and running, and LPA was defined as stationary plus. MVPA minutes and sedentary minutes were then averaged across all valid wear days to produce summary measures of the mean MVPA minutes per day and the mean sedentary minutes per day for each participant.

### 2.5. Data Analysis

Data analyses were conducted with SAS v9.4 (SAS Institute Inc., Cary, NC, USA). Descriptive statistics, including frequencies, proportions, and means, were calculated for all baseline sociodemographic, health, and program (e.g., intervention dose) variables in the total sample. Sociodemographic and health variables were compared across groups using analysis of variance tests for continuous variables and Fisher’s exact tests for categorical variables. Descriptive statistics were used to quantify program participation (e.g., intervention dose) and report mean within-person PA and sedentary behavior changes of children, fathers, and mothers for each subpopulation as a whole, and by randomized group assignment, for both control and intervention periods.

To determine the overall effect of the intervention, statistical analyses of the intervention effect for each outcome variable, daily minutes of MVPA, LPA, and sedentary behavior for the child, father, and mother were conducted using linear mixed models (PROC MIXED, SAS 9.4). Models account for the hierarchical structure of the data, are recommended for use with stepped-wedge cluster designs [53,65], and allow for the analysis of partial datasets with dropouts or missing study visits. Analyses followed intention-to-treat principles [65,66]. In each model, we included a fixed effect for each scheduled time step in the design [53,65].

Modeling the random effects and correlation structure included an analysis of the best model fit for the observed correlations in the data and the overall model fit using information criteria (AIC, BIC) [67]. Random effects for the assigned study group were included in the model to account for non-independence of members in the same intervention group as either a random intercept or random slope. For all outcomes, when both random effects were included, one estimated variance parameter was zero. We included non-independent covariance structures at the subject level to account for repeated measurements on the same individuals. An unstructured correlation was optimal for outcomes with different variance estimates for each time point and different covariances between time points. For all other outcomes, a model with fewer parameters was chosen. Modeling choices were conservative to include retaining outliers in a few instances. Models were all assessed for adequacy and appropriateness of model assumptions.

Figures were created to visualize the group-level mean MVPA, LPA, and sedentary behavior at each data collection time point. Student’s *t*-tests were used to determine the statistical significance (at the α = 0.05 level) of group change in MVPA, LPA, and sedentary behavior.

## 3. Results

Table 1 provides descriptive characteristics of the study sample. In total, 59 families participated in this study. Across all groups, the mean ages were 10.1 years (SD = 0.9) for children, 39.9 years (SD = 8.2) for fathers, and 36.2 years (SD = 6.2) for mothers. In the total sample, 36.2% of children had a healthy BMI-for-age, 32.8% had an overweight BMI-for-age, and 31.0% had an obese BMI-for-age. Approximately half (52.5%) of the children were female. Many fathers (48.3%) and most mothers (65.5%) were obese (BMI ≥ 30) at baseline. There were no significant differences in the BMI for children, fathers, or mothers across groups. Before the intervention, on average, children spent 50.6 min (±17.8) in daily MVPA, 230.2 min (±55.7) in daily LPA, and 619.5 min (±62.5) in daily sedentary behavior; fathers spent 75.1 min (±35.7) in daily MVPA, 547.7 min (±98.4) in LPA, and 373.2 min (±91.5) in daily sedentary behavior.

Approximately 52.5% (n = 31) of families attended all six program sessions; 6.8% (n = 4) attended four to five, 3.4% (n = 2) attended three, 22% (n = 13) attended one to two, and 15.3% (n = 9) attended no sessions. Every session included PA activities, with PA curriculum implemented for an average of 22 min (SD = 15.31) each week (sessions 1–3 and 5: 13 min each; session 4: 50 min; and session 6: 30 min) [48]. Approximately 78.0% (n = 46) of families received 100–132 min of PA-specific programming, 15.2% (n = 9) received 1–99 min, and 6.8% (n = 4) received 0 min. Table 2 describes the program effects on MVPA, LPA, and sedentary behavior. For children, there were no significant changes in MVPA (*p* = 0.18), LPA (*p* = 0.52), or sedentary behavior (*p* = 0.74). For fathers, there were no significant changes in MVPA (*p* = 0.94), LPA (*p* = 0.17), or sedentary behavior (*p* = 0.15). For mothers, there was a *significant* decrease in LPA (*p* < 0.01) and no significant changes in MVPA (*p* = 0.66) or sedentary behavior (*p* = 0.77). Specifically, the program resulted in an average reduction of 23.2 min of LPA among mothers (95% CI: −34.0, −12.4). Table 2 also displays the overall effects on MVPA, LPA, and sedentary behavior after controlling for the number of sessions attended and the number of PA minutes across all attended sessions. After adjustment, findings were consistent with unadjusted models and showed that mothers’ LPA was the only statistically significant outcome impacted by the program. Specifically, the program resulted in an average reduction of 23.1 min (*p* = 0.01) of LPA among mothers after adjusting for the number of sessions attended, and an average reduction of 26.2 min (*p* = 0.02) of LPA among mothers after adjusting for the number of PA minutes across sessions.

**Figure 2:**
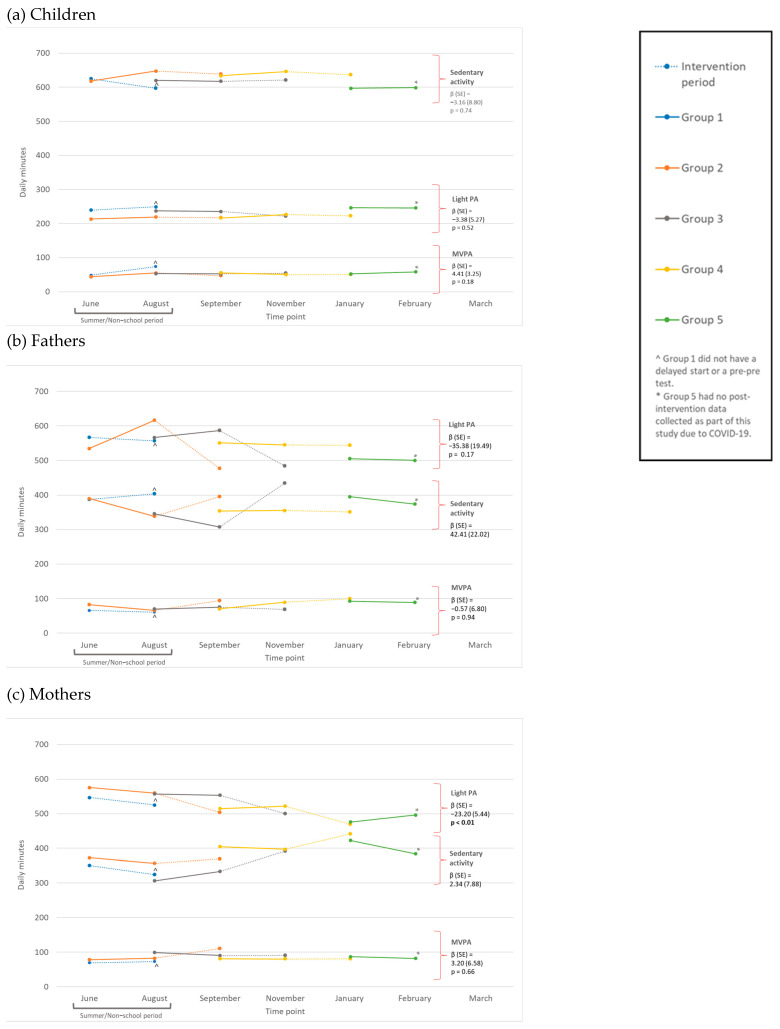
Group-level trial effects on (**a**) children, (**b**) fathers, and (**c**) mothers’ moderate-to-vigorous physical activity (MVPA), light PA (LPA), and sedentary daily minutes. (**b**) Displays changes in daily minutes of MVPA, LPA, and sedentary behavior for *fathers* in each study group. Group 1, who participated in the program during summer/non-school months (unlike other groups), showed non-significant trends for fathers’ MVPA, LPA, and sedentary behavior. Group 2 showed significant decreasing trends for fathers’ LPA and significant increasing trends for MVPA and sedentary behavior (*p* < 0.05). Group 3 showed non-significant trends for fathers’ MVPA and LPA, and significant increasing trends for sedentary behavior (*p* < 0.05). Group 4 showed non-significant trends for fathers’ LPA and sedentary behavior and increasing trends for MVPA. For more information, see Supplementary Information S3.

Figure 2a displays changes in daily minutes of MVPA, LPA, and sedentary behavior for *children* in each study group. Group 1, which received the program during summer/non-school months (unlike other groups), showed non-significant trends for child sedentary behavior during the program, significant increasing trends for MVPA (*p* < 0.05), and non-significant trends for LPA. Group 2 showed significant decreasing trends for child MVPA (*p* < 0.05), and non-significant trends for LPA and sedentary behavior. Group 3 showed non-significant trends for children’s LPA and increasing trends for MVPA and sedentary behavior. Group 4 showed non-significant trends for children’s LPA and sedentary behavior, and almost no changes in MVPA. For more information, see Supplementary Information S2.

Figure 2c displays changes in the daily minutes of MVPA, LPA, and sedentary behavior for *mothers* in each study group. Group 1, who participated in the program during summer/non-school months (unlike other groups), showed non-significant trends for mothers’ LPA, MVPA, and sedentary behavior. Group 2 showed non-significant trends for mothers’ LPA and sedentary behavior, and significant increasing trends for MVPA (*p* < 0.05). Groups 3 and 4 showed non-significant trends for mothers’ LPA, MVPA, and sedentary behavior. For more information, see Supplementary Information S4.

## 4. Discussion

This study described PA and sedentary behavior outcomes for participating Mexican-heritage children, fathers, and mothers in a family-centered, father-focused program titled *¡Haz Espacio para Papi!* (Make Room for Daddy). This paper evaluated program effects on MVPA, LPA, and sedentary behavior. In doing so, this study fills an important gap in the literature on father-focused and family-centered PA programs. Overall, the results demonstrated that the PA program components had no significant, positive impact on MVPA, LPA, or sedentary behavior among children, fathers, or mothers. Although mothers, who engaged in only half of the sessions, as designed (sessions 1, 2, 4; totaling 76 min of PA-focused program time) [48], saw a significant change in LPA from baseline to post-assessment, the effect was negative.

While this study did not show an overall, significant intervention effect on MVPA, LPA, or sedentary behavior outcomes for participants, comparisons between our findings and past research can elucidate reasons for null effects. Past interventions that focused on Hispanic or Mexican-heritage individuals by providing access to, or referrals for, PA resources (in addition to educational materials) showed larger effects [68]. For our study, the program might have been limited in its impact on PA outcomes over time among participants with low access to PA resources, which is shown to be necessary for maintaining regular PA within *colonias* neighborhoods [17]. In addition, community-based interventions have effectively increased walking and decreased depression and stress among Hispanic or Mexican-heritage participants, so it may be that programs focused less on the nuclear family unit and more on larger social networks may be more effective [68,69]. Lastly, this PA program was included as part of a larger program that addressed healthy eating behavior. Past research in this field has largely examined independent PA interventions [68,70,71]. Given the time needed to address healthy eating and cooking demonstrations in the HEPP!, it is likely that the dose of PA instruction and programming (i.e., a total of 132 min across 6 weeks, 14.67% of the total intervention time, where four sessions only had 13 min of PA programming) was too small to have a significant effect.

One program that shows many similarities to the intervention presented in this paper is the “Healthy Dads, Healthy Kids” (HDHK) program developed in Australia, which significantly increased child PA for white fathers and children [47,72]. Results from HDHK-based programs have demonstrated significant PA impacts, which differs from our study results [40,42,69,70,71,72]. To note, HDHK in Australia and as applied in Houston, U.S., sampled fathers or children that were experiencing an overweight or obese weight status at enrollment, so it may be that this type of intervention only yields significant behavioral impact when recruiting participants with low rates of PA and healthy eating at baseline [40,42,69,70,71,72]. Our study did not implement eligibility criteria related to weight status, but 88.0% of fathers and 63.8% of children were overweight or obese in our study population. In addition, HDHK studies showed a higher dose of PA program components than the HEPP!, ranging from seven total face-to-face sessions (90 min/session, 630 min total contact time) [46] to eight total face-to-face sessions (75 min/session, 600 min total contact time) with fathers [47], and a total of 225–270 total PA minutes (e.g., rough and tumble play, fun fitness circuits, and active games). In HEPP!’s six-session program (150 min/session, 900 min total contact time), four sessions (one, two, three, and five) included 13 min of PA; session four included 50 min of PA, and session six included 30 min of PA (total practical PA time = 132 min). Additional contact time focused on cooking demonstrations, healthy eating, and enhancing family dynamics. In addition, HEPP! included 6 weeks of intervention and follow-up measurements at 6 weeks, compared to 7 and 8 weeks of intervention and follow-up measurements at 14 and 24 weeks, as done in the two HDHK studies [46,47,73,74].

### Limitations and Strengths

This study has several limitations. First, the sample size for the study was small, which limited our ability to study group-level intervention outcomes. Therefore, our results are not generalizable to the broader population of Mexican-heritage families living in *colonias.* It should also be noted that this study employed all-female implementation team members (*promotoras*). It is possible that fathers could experience more program connection and engagement with male implementers (*promotores*), which should be considered in future programs. Challenges with participant retention, compliance with the intervention plan, and missing participant data should be considered when interpreting results from this study. Data from the process evaluation, previously reported, show that 24.2% of families did not engage in the weekly take-home challenges for PA [48]. Lastly, we did not measure environmental factors that may influence long-term impacts, such as access to PA resources or neighborhood safety [75,76]. Future research should assess and account for differences in access to safe PA spaces.

This study also has strengths. First, HEPP! is a theory-informed program that incorporates family and culturally inclusive elements. To date, no existing research has implemented a father-focused and family-centered PA program among Mexican-heritage residents of *colonias* to address low levels of PA among children and adults [19]. Second, we used accelerometers to measure PA, which addresses bias that is often present in research using self-reported measures of PA among children and adults [77]. Lastly, we used a modified stepped-wedge design to maximize the benefit to participating families and reduce resources needed to provide the program, while also providing a control group. Similar study designs should be considered in future research to ensure that positive impacts of health programs are accessible to all participants, in turn, potentially improving community acceptance of the program [53].

## 5. Conclusions

These study findings have important research implications. First, since past research shows that family-based interventions can increase father–child bonding and behavior reinforcement [43,46,47], future research should examine whether social (e.g., social support) and self-regulatory (e.g., self-efficacy, skill competency) outcomes are the mechanisms through which the program influences PA [78]. Second, additional research is needed to investigate the impact of family-centered interventions on replacement activity to understand whether the intervention increases PA or simply replaces existing PA behaviors with new ones. Third, research is needed that increases the dose of PA interventions and has longer follow-up periods to understand whether HEPP! can positively impact PA. Examinations of dose response within PA interventions like HEPP! may be needed to determine the optimal duration and frequency for such family-centered father-focused programs. Finally, future research on the impact of family-centered interventions should quantify and account for PA barriers that fathers and families face (e.g., time constraints) [73,74]. While PA barriers were measured in our study, they were collected in an open-ended qualitative format which did not allow for statistical adjustment.

This study has additional implications for public health practice. Although we presented models adjusting for the number of sessions attended and the number of minutes of PA-focus across all sessions, and saw no differences compared to unadjusted models, null findings may indicate that the overall dose of PA components was too small (max PA-focused minutes = 132 min across six sessions). Future family-centered programs should consider increasing the dose of PA-focused components when coupled with healthy eating strategies by implementing PA components for a longer duration within program sessions, running the program for longer periods of time (e.g., >3 months), as well as increasing follow-up periods (e.g., >1 year) to assess potential long-term changes [46,47,68]. Evaluating the necessary dose for both PA and healthy eating components, when paired with a family-centered approach, should be prioritized. Next, integrating cultural needs and preferences of the priority population are crucial, especially when low-income groups or communities experiencing marginalization are being engaged. Family-centered programming, based on a *promotora* model, showed promise in supporting Mexican-heritage families in a marginalized community. Future programming efforts should apply approaches, curricula, or strategies to tailor programs for underserved populations.

## Figures and Tables

**Table 1:** Baseline Characteristics of Randomized Families, for Total Sample and by Group Assignment.

	Total	Group 1 ^1^	Group 2	Group 3	Group 4	Group 5	*p* ^2^
Number of families: n	59	12	10	13	12	12	
Age: mean years (SD)							
Child	10.1 (0.9)	10.2 (0.9)	10.3 (0.9)	10.1 (1.0)	10.5 (0.9)	9.7 (0.9)	0.31
Father	39.9 (8.2)	39.8 (11.8)	40.9 (7.1)	39.4 (7.5)	38.0 (6.5)	42.1 (7.8)	0.82
Mother	36.2 (6.2)	35.2 (7.3)	37.2 (6.2)	37.0 (5.2)	35.1 (5.7)	36.5 (7.1)	0.90
Child sex: n (%)							
Female	52.5	58.3	70.0	23.1	66.7	50.0	0.14
Male	47.5	41.7	30.0	76.9	33.3	50.0	
Moderate-to-vigorous physical activity: mean daily minutes (SD)							
Child	50.6 (17.8)	48.8 (14.8)	43.9 (19.3)	53.0 (18.0)	55.2 (22.8)	52.2 (12.6)	0.34
Father	75.1 (35.7)	65.6 (23.8)	82.2 (53.7)	69.8 (26.3)	70.2 (25.6)	92.5 (43.7)	0.01 ^3^
Mother	86.4 (36.9)	82.7 (36.1)	78.4 (19.8)	98.7 (51.0)	81.0 (41.8)	86.9 (24.2)	0.09
Light physical activity: mean daily minutes (SD)							
Child	230.2 (55.7)	239.4 (48.0)	213.3 (67.1)	237.2 (52.4)	216.6 (67.0)	246.4 (39.4)	0.25
Father	547.7 (98.4)	567.6 (92.1)	535.3 (103.6)	566.8 (94.5)	551.4 (114.6)	505.8 (88.4)	0.34
Mother	535.3 (86.1)	546.7 (91.0)	575.7 (82.6)	556.8 (90.5)	514.7 (76.6)	475.7 (62.6)	0.05
Sedentary behavior: mean daily minutes (SD)							
Child	619.5 (62.5)	624.9 (51.1)	617.9 (77.7)	619.8 (41.4)	633.9 (91.2)	597.1 (32.0)	0.20
Father	373.2 (91.5)	387.6 (58.2)	390.1 (86.8)	345.6 (93.8)	354.1 (107.6)	395.4 (112.0)	0.46
Mother	369.5 (85.8)	350.5 (89.8)	372.7 (57.7)	306.2 (67.9)	404.7 (83.7)	422.5 (86.0)	<0.01 ^4^

^1^ Group 2 estimates for moderate-to-vigorous physical activity, light physical activity, and sedentary behavior were recorded at time point 2 (instead of baseline). ^2^ Analysis of variance tests were used for continuous variables, and Fisher’s Exact Test was used for categorical variables to test their differences between groups. ^3^ Statistically significant difference between Group 1 and Group 4, and Group 1 and Group 5 only. ^4^ Statistically significant difference between Group 1 and Group 4, and Group 3 and Group 4 only.

**Table 2:** Trial Effects on Child, Father, and Mother’s Moderate-to-Vigorous Physical Activity (MVPA), Light Physical Activity (LPA), and Sedentary Behavior Daily Minutes.

	Estimate (Standard Error) *	*p*-Value *
Unadjusted models		
Child outcomes		
MVPA	4.41 (3.25)	0.18
LPA	−3.38 (5.27)	0.52
Sedentary behavior	−3.16 (8.80)	0.74
Father outcomes		
MVPA	−0.57 (6.80)	0.94
LPA	−35.39 (19.49)	0.17
Sedentary behavior	42.41 (22.02)	0.15
Mother outcomes		
MVPA	3.20 (6.58)	0.66
LPA	−23.20 (5.44)	<0.01
Sedentary behavior	2.34 (7.88)	0.77
Models adjusted for number of sessions attended		
Child outcomes		
MVPA	4.38 (4.89)	0.44
LPA	−1.13 (5.74)	0.86
Sedentary behavior	−4.02 (9.35)	0.70
Father outcomes		
MVPA	0.31 (6.91)	0.97
LPA	−35.12 (21.80)	0.21
Sedentary behavior	41.17 (24.73)	0.19
Mother outcomes		
MVPA	3.04 (7.26)	0.70
LPA	−23.09 (4.40)	0.01
Sedentary behavior	16.47 (20.33)	0.50
Models adjusted for total number of physical activity minutes across sessions		
Child outcomes		
MVPA	4.24 (4.73)	0.44
LPA	−1.40 (6.18)	0.84
Sedentary behavior	−5.06 (8.55)	0.60
Father outcomes		
MVPA	−0.03 (7.34)	0.99
LPA	−37.32 (19.10)	0.15
Sedentary behavior	40.41 (22.83)	0.17
Mother outcomes		
MVPA	3.08 (7.36)	0.70
LPA	−26.24 (5.22)	0.02
Sedentary behavior	2.24 (7.81)	0.79

Moderate-to-vigorous physical activity (MVPA), light physical activity (LPA), and sedentary behavior. * Model estimates and related significance account for non-independence between measurements for the same participant and include random intercepts and slopes for group-level effects.

## Data Availability

The datasets used and/or analyzed during the current study are available from the corresponding author on reasonable request.

## Supplementary Materials

The following supporting information can be downloaded at: https://www.mdpi.com/article/10.3390/ijerph21111475/s1, Figure S1. Visualization of the ¡Haz Espacio para Papi! (HEPP!) Stepped-Wedge Study Design. Table S1: Participation and outcome characteristics of children, for all children and by group assignment. Table S2: Participation and outcome characteristics of fathers, for all fathers and by group assignment. Table S3. Participation and outcome characteristics of mothers, for all mothers and by group assignment.
