# Supplementary material for: Physical Activity Outcomes of a Culturally Tailored, Father-Focused, and Family-Centered Health Promotion Program for Mexican-Heritage Families: ¡Haz Espacio Para Papi! (Make Room for Daddy)
Source: Int J Environ Res Public Health. 2024 Nov 6;21(11):1475. doi: 10.3390/ijerph21111475 (PMC11593588; doi:10.3390/ijerph21111475)
Supplement: Supplementary file 1 [file ijerph-21-01475-s001.zip › ijerph-3205900-supplementary.pdf]

Supplemental Document S1. Visualization of the *¡Haz Espacio para Papi!* (HEPP!) Stepped-Wedge Study Design.

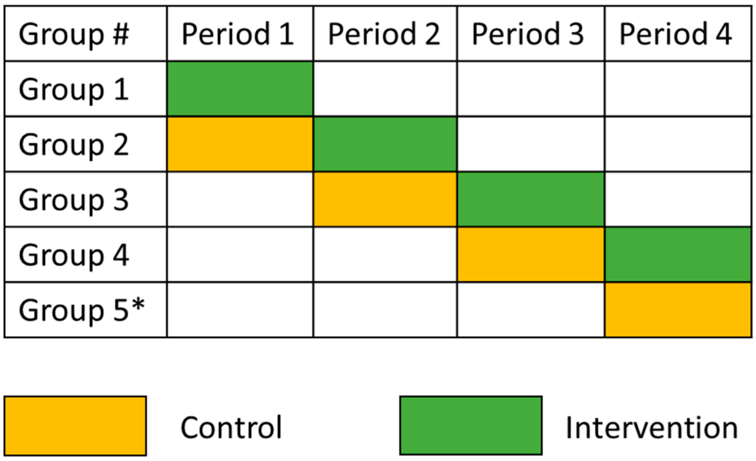

Supplemental Document S2. Participation and outcome characteristics of children, for all children and by group assignment.

	Total	Group 1	Group 2	Group 3	Group 4	Group 5
**CONTROL PERIOD**						
Complete data, n	41	---	10	11	12	8
Within-person Δ Sedentary Time, minutes (SD)	14.1 (95.1)	---	11.4 (86.2)	18.6 (92.5)	12.2 (114.2)	14.1 (96.7)
Within-person Δ LPA, minutes (SD)	0.6 (94.7)	---	26.8 (79.0)	-22.9 (92.8)	10.0 (89.3)	-13.8 (126.9)
Within-person Δ MVPA, minutes (SD)	1.4 (26.3)	---	15.7 (25.4)	-2.2 (22.7)	-4.8 (31.9)	-2.1 (19.8)
Intervention dose, number of sessions (SD)	4.6 (2.3)	4.3 (2.4)	4.1 (2.6)	4.5 (2.5)	5.4 (1.7)	---
**INTERVENTION PERIOD**						
Complete data, n	40	9	8	11	12	---
Within-person Δ Sedentary Time, minutes (SD)	-12.4 (99.9)	-42.8 (98.0)	11.3 (123.9)	-7.6 (113.3)	-9.7 (75.8)	---
Within-person Δ LPA, minutes (SD)	-6.2 (71.8)	18.3 (77.7)	-21.4 (87.8)	-18.0 (83.2)	-3.8 (43.1)	---
Within-person Δ MVPA, minutes (SD)	4.7 (28.1)	28.4 (24.7)	-12.8 (11.9)	3.0 (33.1)	0.3 (23.5)	---

MVPA = moderate-to-vigorous physical activity, LPA = light physical activity; Δ = change, SD = standard deviation.

Supplemental Document S3. Participation and outcome characteristics of fathers, for all fathers and by group assignment.

	Total	Group 1	Group 2	Group 3	Group 4	Group 5
**CONTROL PERIOD**						
Complete data, n	41	---	10	12	12	7
Within-person Δ Sedentary Time, minutes (SD)	-31.8 (123.9)	---	-35.9 (102.2)	-54.7 (128.8)	1.1 (146.8)	-42.8 (115.8)
Within-person Δ LPA, minutes (SD)	30.8 (132.7)	---	53.4 (113.7)	49.7 (165.4)	-5.9 (143.3)	29.1 (77.1)
Within-person Δ MVPA, minutes (SD)	2.4 (44.6)	---	-2.2 (21.6)	-6.9 (61.4)	18.9 (40.0)	-4.1 (43.9)
Intervention dose, number of sessions (SD)	4.6 (2.3)	4.3 (2.4)	4.1 (2.6)	4.5 (2.5)	5.4 (1.7)	---
**INTERVENTION PERIOD**	40	9	9	10	12	
Complete data, n						---
Within-person Δ Sedentary Time, minutes (SD)	50.2 (103.5)	31.5 (86.9)	65.1 (74.7)	119.0 (124.5)	-4.4 (87.9)	---
Within-person Δ LPA, minutes (SD)	-63.1 (117.2)	-24.2 (54.7)	-151.9 (92.0)	-92.1 (143.0)	-1.4 (104.9)	---
Within-person Δ MVPA, minutes (SD)	7.0 (33.0)	-4.3 (29.4)	33.1 (28.4)	-10.4 (33.9)	10.2 (27.5)	---

MVPA = moderate-to-vigorous physical activity, LPA = light physical activity, Δ = change, SD = standard deviation.

Supplemental Document S4. Participation and outcome characteristics of mothers, for all mothers and by group assignment.

	Total	Group 1	Group 2	Group 3	Group 4	Group 5
**CONTROL PERIOD**						
Complete data, n	43	---	10	13	12	8
Within-person Δ Sedentary Time, minutes (SD)	-5.2 (110.8)	---	-9.1 (54.0)	18.7 (89.0)	-7.8 (156.5)	-35.1 (125.3)
Within-person Δ LPA, minutes (SD)	-2.2 (95.6)	---	-24.7 (82.3)	2.5 (64.5)	7.2 (135.5)	4.1 (95.9)
Within-person Δ MVPA, minutes (SD)	-2.4 (40.1)	---	2.8 (40.1)	-6.5 (36.8)	-0.2 (51.3)	-5.5 (32.5)
Intervention dose, number of sessions (SD)	4.6 (2.3)	4.3 (2.4)	4.1 (2.6)	4.5 (2.5)	5.4 (1.7)	---
**INTERVENTION PERIOD**						
Complete data, n	42	9	9	12	12	---
Within-person Δ Sedentary Time, minutes (SD)	24.7 (98.9)	-20.2 (51.2)	9.9 (91.2)	49.9 (81.4)	44.1 (137.7)	---
Within-person Δ LPA, minutes (SD)	-43.6 (125.8)	-16.5 (131.0)	-56.1 (149.8)	-46.1 (127.9)	-52.2 (113.9)	---
Within-person Δ MVPA, minutes (SD)	6.1 (34.0)	-0.1 (22.3)	24.8 (25.6)	2.5 (45.3)	0.3 (32.4)	---

MVPA = moderate-to-vigorous physical activity, LPA = light physical activity, Δ = change, SD = standard deviation.
